# Comparative effectiveness of novel laser-assisted technologies for treating peri-implantitis: A network meta-analysis of randomized controlled trials

**DOI:** 10.1097/MD.0000000000051256

**Published:** 2026-10-30

**Authors:** Zhe Wang, Jing Wen, Haoyang Li, Jiaying Xu, Furong Mao

**Affiliations:** a Department of Oral and Maxillofacial Surgery, Wuxi Stomatology Hospital, Wuxi, Jiangsu Province, China; b Wanshun Outpatient Department, Wuxi Stomatology Hospital, Wuxi, Jiangsu Province, China; c Department of Periodontics, Wuxi Stomatology Hospital, Wuxi, Jiangsu Province, China.

**Keywords:** clinical outcomes, dental implants, laser therapy, network meta-analysis, peri-implantitis, photodynamic therapy

## Abstract

**Background:**

Peri-implantitis impairs dental implant survival. Though new laser-assisted therapies are promising, their relative efficacy remains unclear. This Bayesian network meta-analysis compared photodynamic therapy (PDT), diode laser (DL), erbium:YAG laser (Er:YAG), and conventional non-laser control (CON).

**Methods:**

Following PRISMA guidelines, we retrieved relevant randomized controlled trials from PubMed, Embase, Web of Science, MEDLINE, and China National Knowledge Infrastructure up to May 31, 2025. Primary outcomes included bleeding on probing (BOP), probing depth (PD), and plaque index; secondary outcomes covered clinical attachment level, crestal bone loss, gingival index, and bleeding index (BI). We calculated odds ratios and standardized mean differences with 95% credible intervals and *P*-values via Stata 14. Treatments were ranked using the surface under the cumulative ranking curve, combined with effect estimates and evidence quality for result interpretation.

**Results:**

Thirty-three randomized controlled trials with 1825 participants were included. PDT ranked best overall, with significant improvements in PD, plaque index, crestal bone loss, gingival index, and BI versus CON. No therapy yielded significant differences in BOP and clinical attachment level. DL showed no clinical advantages over CON, while Er:YAG only improved BI significantly. Surface under the cumulative ranking curve rankings of DL and Er:YAG only indicated probability order, not definite therapeutic effects.

**Conclusion:**

PDT exerted the most stable short-term efficacy for peri-implantitis. DL and Er:YAG had limited valid benefits. Due to insufficient long-term data, the sustained effects of these treatments and their roles in preventing lesion recurrence and maintaining implant survival are still undetermined. A network meta-analysis of 33 RCTs found that photodynamic therapy (PDT) had the most consistent statistically supported benefits, with significant improvements versus CON in PD, PI, CBL, GI, and BI, but not in BOP or CAL. Although PDT also showed the most favorable SUCRA based ranking profile, SUCRA reflects ranking probability rather than effect magnitude, certainty of evidence, or definitive clinical superiority. DL did not show statistically significant superiority over CON, and Er:YAG showed a significant benefit versus CON only for BI. These findings support considering PDT as a minimally invasive adjunct to mechanical debridement when clinically appropriate, while emphasizing that treatment selection should be guided by statistically robust effects, CrI precision, patient-specific factors, and the limitations of the underlying evidence network. Importantly, the current evidence mainly reflects short term clinical response. Whether PDT provides sustained disease control, reduces recurrence, or improves long term implant survival remains to be confirmed in well powered trials with longer follow-up.

## 1. Introduction

Peri-implantitis is defined by the 2018 World Workshop on the Classification of Periodontal and Peri-Implant Diseases and Conditions as bleeding and/or suppuration on gentle probing, increased probing depth (PD) compared to previous examinations, and bone loss beyond initial crestal remodeling.^[1]^ Peri-implantitis is a clinically important complication of dental implants, characterized by inflammation of the peri-implant soft tissues and progressive bone loss. Recent pathological evidence further indicates that peri-implantitis lesions may involve progressive connective tissue degradation and alveolar bone resorption, supporting its destructive inflammatory nature.^[2]^ The pathogenesis of peri-implantitis is multifactorial, involving microbial infection, host immune response, and the properties of the implant surface.^[3]^ If not treated in a timely manner, peri-implantitis may lead to implant failure and cause discomfort, functional impairment, and psychological distress. Given its potential impact on oral function and implant survival, early diagnosis and effective management remain essential.^[4]^

Currently, the common treatment modalities for peri-implantitis include mechanical debridement, chemical therapy, and surgical interventions.^[3,5]^ Mechanical debridement typically involves the use of ultrasonic scalers or manual curettes to remove biofilm and plaque from the implant surface. However, mechanical debridement alone may not completely eliminate pathogenic bacteria and can potentially damage the implant surface if not performed correctly. Chemical therapy, such as the use of chlorhexidine, helps reduce bacterial load but has limited efficacy and may cause adverse effects on oral mucosa with prolonged use.^[6]^ Surgical intervention can be effective in advanced cases, but it is more invasive and is associated with longer recovery. Consequently, there is continued interest in effective, adjunctive, and minimally invasive treatment options.^[7]^

Laser technology has emerged as a promising adjunctive treatment in peri-implantitis management, showing significant advantages when combined with mechanical debridement. Laser-assisted approaches may contribute to microbial reduction, biofilm control, and tissue decontamination in a minimally invasive manner, although their clinical effects depend on wavelength, energy delivery, irradiation mode, and treatment protocol.^[8]^ Novel laser technologies such as CO_2_ lasers, semiconductor lasers like diode lasers (DLs), and erbium lasers such as Er:YAG and Er,Cr:YSGG have been investigated for their potential roles in reducing inflammation, supporting soft and hard tissue healing, and improving postoperative comfort.^[9,10]^ Clinical studies and reviews have also reported beneficial effects of laser-assisted or photobiomodulation-based therapies across dental and peri-implant outcomes, but the magnitude and durability of these effects remain protocol-dependent.^[11,12]^ However, reported effects vary across studies because of differences in study design, sample size, laser parameters, follow-up duration, and outcome definitions. Consistent with this concern, the recent World Federation for Laser Dentistry consensus report emphasized the need for standardized parameters, careful safety control, and evidence-based interpretation of laser-assisted procedures in dentistry.^[13]^ Therefore, the comparative effectiveness of different laser-assisted modalities remains uncertain.^[14]^ Previous systematic reviews and network meta-analyses have examined select laser comparisons – such as photodynamic therapy (PDT) versus Er:YAG or DLs versus conventional therapy – but these have been limited to pairwise analyses or subsets of modalities.

Several pairwise systematic reviews have compared PDT with Er:YAG or conventional treatment, but few studies have simultaneously compared PDT, DL, Er:YAG, and non-laser approaches across multiple clinical and radiographic outcomes within a unified evidence network. To address this gap, this study conducted a Bayesian network meta-analysis of randomized controlled trials (RCTs) to evaluate the comparative effectiveness of novel laser-assisted technologies in the treatment of peri-implantitis. In addition to estimating treatment effects with 95% credible intervals, we used surface under the cumulative ranking curve (SUCRA) values as a supplementary ranking metric to describe the probability of each intervention occupying a favorable rank. These rankings were interpreted alongside effect size, statistical precision, and the directness of evidence, rather than as independent proof of clinical superiority. By integrating direct and indirect evidence, this study aims to provide a more balanced evidence base for clinical decision-making in peri-implantitis management.

## 2. Materials and methods

This review protocol was prospectively registered in PROSPERO (CRD420251079476) and follows the PRISMA guidelines.^[15]^ Our research question was: “In adults with peri-implantitis, what is the comparative effectiveness of PDT, DL, Er:YAG laser, and conventional non-laser treatments on clinical and radiographic outcomes?”

### 2.1. Search strategy

We conducted a comprehensive search from database inception to May 31, 2025, across PubMed, MEDLINE, Embase, Web of Science, and the China National Knowledge Infrastructure, without applying language restrictions. Search strategies for Embase, Web of Science, MEDLINE, and China National Knowledge Infrastructure are detailed in Supplementary File 1, Supplemental Digital Content 1. Additionally, manual searches of the reference lists of relevant articles and reviews were performed to identify further studies. Title/abstract screening and full-text review were independently conducted by 2 researchers. Any discrepancies were resolved through discussion and, if necessary, by consulting a third author.

### 2.2. Inclusion and exclusion criteria

The inclusion criteria for this systematic review and network meta-analysis were as follows: studies involving adults aged 18 and above diagnosed with peri-implant diseases, including peri-implant mucositis and peri-implantitis; intervention groups using laser-assisted technologies for peri-implantitis treatment, with interventions classified into clinically prespecified modality nodes according to the reported laser type and principal mechanism of action, including PDT, DL, and Er:YAG; control groups receiving traditional mechanical debridement, chemical treatments, or other nonsurgical treatments not involving laser technologies; and studies reporting at least 1 outcome, such as clinical attachment loss (CAL), PD, bleeding on probing (BOP), or gingival index (GI). This node structure was used to preserve clinically meaningful distinctions between photosensitizer-mediated PDT, semiconductor laser-based therapy, and erbium laser-based decontamination or ablation, while allowing comparison across trials with heterogeneous protocols. Only randomized controlled trials (RCTs) with a minimum follow-up period of 3 months were included. Exclusion criteria comprised reviews, case reports, case series, conference abstracts, technical reports, studies with incomplete or duplicate data, and those failing to clearly describe the type of laser technology used or providing incomplete or unextractable outcome data.

### 2.3. Data extraction

Following the identification of all relevant articles from the designated databases, references were systematically managed using EndNote X9. Data extraction for studies meeting the inclusion criteria was independently performed by 2 researchers. To ensure accuracy and reliability, any discrepancies were resolved through consensus among all authors. Extracted data included basic publication information such as author names, titles, years, and journals, as well as patient demographics (age, gender), disease type, treatment interventions, and outcomes. When available, laser-specific protocol details were also extracted, including wavelength, power or energy density, photosensitizer type, irradiation time, number of treatment sessions, and adjunctive protocols such as mechanical debridement or chlorhexidine use. For PD, CAL, and GI, we extracted mean change values calculated as posttreatment minus baseline; when only endpoint values were reported, change scores and their standard deviations were derived following Cochrane Handbook methods. If essential data were missing from primary studies, attempts were made to contact the authors at least 4 times within 6 weeks to obtain the necessary information.

### 2.4. Outcomes

The primary outcomes of interest included BOP, PD, plaque index (PI), clinical attachment level (CAL), crestal bone loss (CBL), gingival index (GI), and bleeding index (BI). These outcomes were chosen to comprehensively evaluate the effectiveness of various laser technologies in treating peri-implantitis by assessing both clinical and radiographic measures. All continuous outcomes were analyzed as change scores (posttreatment minus baseline) to ensure consistency across trials.

### 2.5. Risk of bias assessment

The risk of bias in the included studies was assessed at the study level using the revised Cochrane risk-of-bias tool (RoB 2).^[16]^ This assessment covered the following domains: the randomization process, deviations from intended interventions, missing outcome data, measurement of outcomes, and selection of the reported result. Any disagreements in the assessments were resolved through consultation with a third reviewer, ensuring a rigorous and unbiased evaluation of the included studies.

### 2.6. Data synthesis

Data synthesis was performed using Stata software (version 14, StataCorp LLC) to generate network diagrams that visually represent the network of trial comparisons, ensuring the feasibility of the network meta-analysis. A Bayesian network meta-analysis was conducted using the “network” and “mvmeta” packages in Stata to compare the effects of different types and doses of laser interventions. Effect sizes for dichotomous outcomes were calculated as odds ratios (ORs), while effect sizes for continuous outcomes were calculated as mean differences (MDs). All continuous outcomes were subsequently standardized to standardized mean differences due to the use of different scales and units in assessing PD, CAL, and CBL. The reliability of the estimates was assessed using 95% credible intervals (CrIs). A random-effects model was employed to pool the data, with non-informative normal priors (mean = 0, variance = 10,000) for treatment effects and a uniform prior (0, 5) for the between-study standard deviation (τ). Markov Chain Monte Carlo sampling was performed with 4 chains, each running 1,50,000 iterations, discarding the first 50,000 as burn-in, and thinning every 10th iteration. Convergence was confirmed using the Gelman–Rubin potential scale reduction factor (PSRF < 1.05 for all parameters). For network construction, laser-assisted interventions were grouped as PDT, DL, and Er:YAG according to their reported modality and main mechanism of action. This approach allowed modality-level comparison while preserving the main clinical distinctions among photosensitizer-mediated therapy, semiconductor laser therapy, and erbium laser-based decontamination or ablation.

Before interpreting the network estimates, we assessed the key assumptions of network meta-analysis. Transitivity was examined by comparing major clinical and methodological characteristics across treatment comparisons, including patient population, intervention type, control treatment, follow-up duration, and outcome definitions. Consistency was further assessed using a global inconsistency test and node-splitting analysis where both direct and indirect evidence were available. A *P* value below .05 was considered suggestive of inconsistency. The results of these analyses are presented in Supplementary Files 2 and 3, Supplemental Digital Content 2 and 9. To explore protocol-related heterogeneity, exploratory univariable meta-regression was performed for BOP, PD, and PI using laser modality, follow-up duration per 3 months, and CHX-containing adjunctive protocol as study-level moderators. Detailed parameter-level analyses based on wavelength, power, energy density, photosensitizer type, irradiation time, or treatment sessions were not performed because these variables were inconsistently reported across trials. The meta-regression results are presented in Supplementary File 4, Supplemental Digital Content 10.

Because most continuous outcomes were synthesized as SMDs, statistically significant findings were also interpreted in relation to clinical relevance. Since universally accepted MCID thresholds for peri-implantitis outcomes are not established, clinical interpretation considered the direction and precision of effect estimates, statistical significance, and the clinical role of each outcome. When absolute interpretation was possible, an approximate 1 mm reduction in PD was regarded as potentially meaningful in peri-implantitis management. The certainty of evidence for key network comparisons versus CON was assessed using a GRADE approach adapted for network meta-analysis. Randomized evidence was initially rated as high certainty and was downgraded when concerns were identified in risk of bias, inconsistency, indirectness, imprecision, or publication bias. These judgments were informed by the RoB 2 assessment, network geometry, global and local inconsistency analyses, CrI precision, and funnel plot findings. The final certainty of evidence was rated as high, moderate, low, or very low and is presented in Supplementary File 5, Supplemental Digital Content 11.

The SUCRA was calculated to summarize the probability of each intervention occupying a favorable rank. SUCRA was treated as a supplementary ranking measure and was not interpreted as a direct measure of effect size, statistical significance, certainty of evidence, or clinical superiority. Primary interpretation was based on the network effect estimates, 95% CrIs, statistical significance, and whether the evidence was supported by direct comparisons or mainly by indirect evidence. Clinical interpretation further considered whether statistically significant effects were relevant to disease control, inflammation reduction, plaque control, attachment stability, or bone preservation. Particular caution was applied when interpreting rankings from sparse networks, wide CrIs, or comparisons with limited direct evidence. Potential publication bias was evaluated using adjusted funnel plots, and statistical heterogeneity among the studies was quantified with τ^2^ and *I*^2^ statistics.^[17]^ Egger’s test was used to detect potential publication bias when *P* < .05.

## 3. Results

### 3.1. Characteristics of included studies

The initial electronic search identified 1533 records. After removing duplicates, 355 records were screened by title and abstract, and 63 full-text articles were assessed. Ultimately, 33 studies met the inclusion criteria and were included in the meta-analysis (Fig. 1), involving a total of 1825 participants. Missing outcome data for 3 studies were obtained through direct correspondence with the original investigators, ensuring the completeness of our dataset. Detailed characteristics of the included studies, including participant demographics, treatment modalities, and outcome measures, are systematically presented in Supplementary File 6, Supplemental Digital Content 12. Follow-up duration varied across trials, but most studies reported short-term outcomes, commonly around 3 months, and only a limited number of trials extended beyond 12 months. Therefore, the pooled estimates should primarily be interpreted as short-term treatment responses.

**Figure 1.**
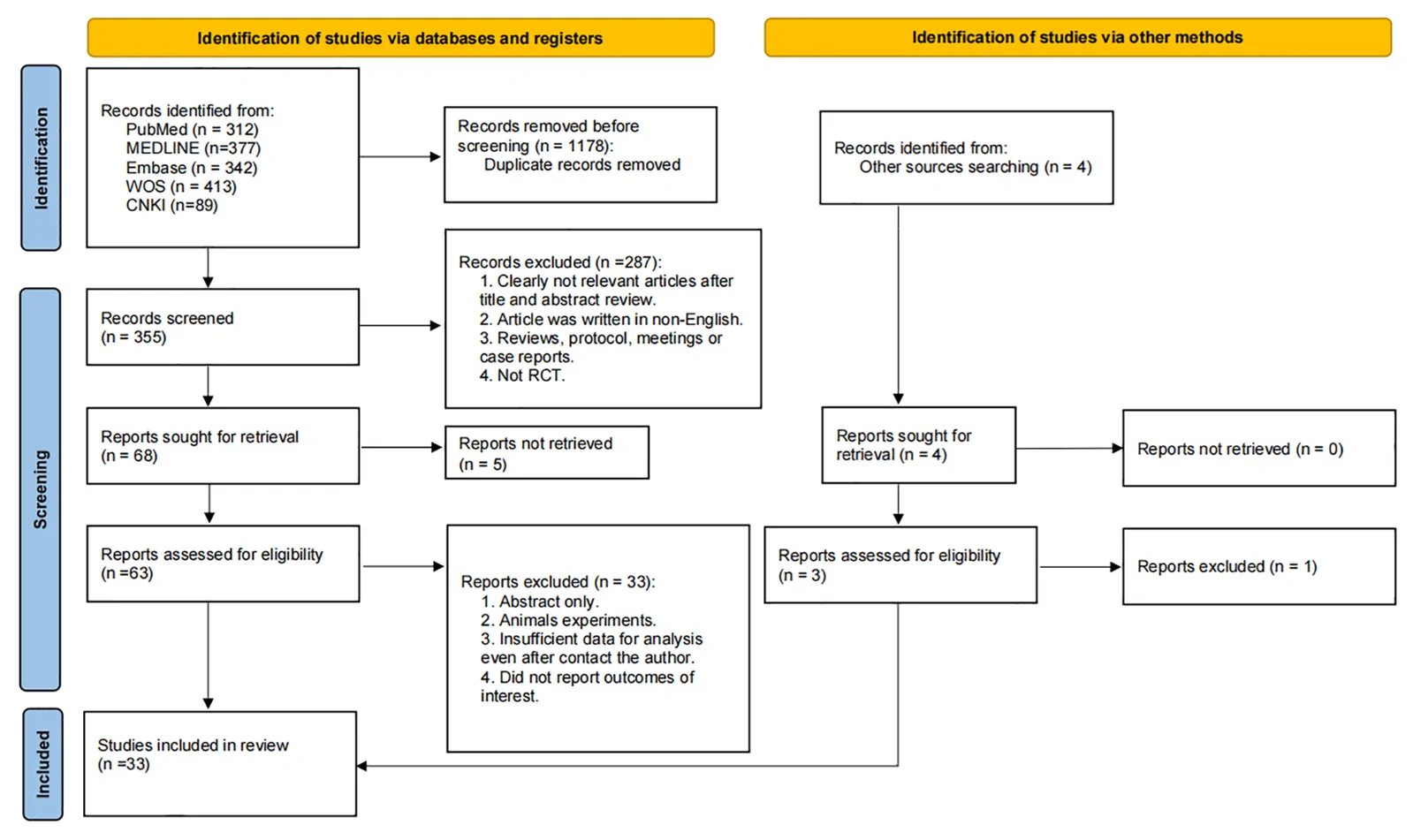
PRISMA flow diagram of the search process for studies. CNKI = China National Knowledge Infrastructure, RCT = randomized controlled trial, WOS = World of Science.

### 3.2. The results of network meta-analysis

Figure 2 depicts the geometry of direct comparisons among the 4 interventions (CON, PDT, DL, and Er:YAG laser) across the 7 clinical endpoints. In every network plot, the CON node is the largest, reflecting its frequent use as the comparator in head-to-head trials. The thickest edge connects CON and PDT, indicating greater precision and more direct evidence for this comparison. In contrast, the edges linking CON to DL and CON to Er:YAG are thinner, indicating fewer direct comparisons. Notably, there were no direct comparisons between DL and Er:YAG or between DL and PDT. Consequently, effect estimates for these pairings relied partly on indirect evidence through CON. Therefore, ranking results for comparisons with limited direct evidence should be interpreted cautiously and should be considered together with the corresponding effect estimates and 95% CrIs, rather than as independent evidence of superiority. To assess statistical heterogeneity, we calculated *I*^2^ and τ^2^ for each outcome network: *I*^2^ ranged from 28% to 62% and τ^2^ from 0.12 to 0.45, indicating moderate heterogeneity across analyses. The included trials were broadly comparable in clinical setting, intervention category, control treatment, and outcome domains, supporting the plausibility of transitivity. However, variation in laser parameters, adjunctive protocols, and follow-up duration was noted and considered when interpreting indirect comparisons.

**Figure 2.**
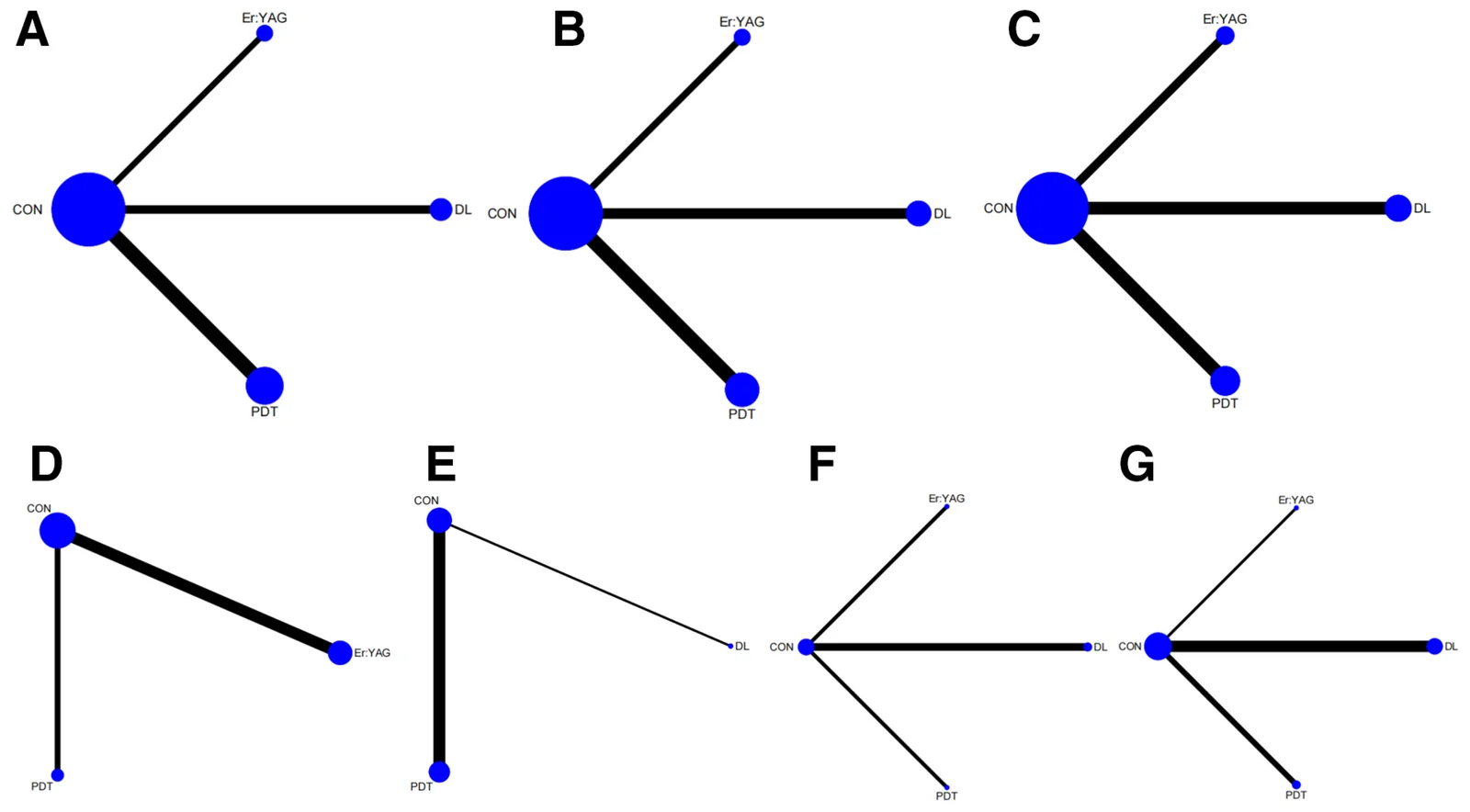
Network of eligible treatment comparisons for (A) BOP, (B) PD, (C) PI, (D) CAL, (E) CBL, (F) GI, (G) BI. Node size is proportional to the number of RCTs involving that intervention; larger nodes indicate more evidence. Edge thickness reflects the inverse variance (precision) of each direct comparison; thicker edges denote comparisons with greater sample size and lower variance. BI = bleeding index, BOP = bleeding on probing, CAL = clinical attachment level, CBL = crestal bone loss, CON = conventional non-laser control, Er:YAG = erbium:YAG laser , GI = gingival index, PD = probing depth, PDT = photodynamic therapy, PI = plaque index, RCT = randomized controlled trial.

#### 3.2.1. Bleeding on probing

In the network meta-analysis for BOP, 24 studies were analyzed to evaluate the effects of different laser-assisted technologies on BOP incidence in patients with peri-implantitis. Figure 2A illustrates the direct comparisons and sample size distribution among the interventions. Results in Table 1 and Figure 3A indicate that no laser modality differed significantly from CON (all *P* > .05). The SUCRA ranking for BOP placed PDT (69.1%), DL (61.7%), and Er:YAG (56.2%) above CON (13.0%). Because none of the pairwise comparisons with CON reached statistical significance, these rankings should be regarded as probabilistic ordering only and not as evidence of confirmed BOP reduction.

**Table 1:** League table of outcomes.

BOP			
PDT			
0.93 (0.19, 4.63)	DL		
0.85 (0.16, 4.53)	0.92 (0.14, 6.00)	Er:YAG	
0.46 (0.18, 1.19)	0.49 (0.14, 1.78)	0.54 (0.14, 2.11)	CON
PD			
PDT			
−0.81 (−2.16, 0.54)	DL		
−0.98 (−2.53,0.58)	−0.16 (−1.80, 1.47)	Er:YAG	
−**1.41** (−**2.30,-0.52**)	−0.59 (−1.61, 0.42)	−0.43 (−1.71, 0.85)	CON
PI			
PDT			
−0.27 (−1.34, 0.79)	DL		
−0.41 (−1.59, 0.77)	−0.14 (−1.36, 1.08)	Er:YAG	
−**0.75** (−**1.47,** −**0.03**)	−0.48 (−1.27, 0.31)	−0.34 (−1.27, 0.59)	CON
CAL			
PDT			
−0.46 (−1.23, 0.31)	Er:YAG		
−0.59 (−1.20, 0.03)	−0.13 (−0.58, 0.32)	CON	
CBL			
PDT			
−**0.33 (-0.55,** −**0.10**)	CON		
−**1.06 (-1.69,** −**0.43**)	−**0.73** (−**1.32, -0.14**)	DL	
GI			
PDT			
−**15.49** (−**19.75,** −**11.24**)	DL		
−**15.55** (−**19.85,** −**11.25**)	−0.06 (−1.04, 0.92)	Er:YAG	
−**15.68** (−**19.90,** −**11.46**)	−0.18 (−0.72, 0.35)	−0.12 (−0.94, 0.69)	CON
BI			
PDT			
−0.12 (−1.42, 1.19)	Er:YAG		
−1.01 (−2.05, 0.03)	−0.89 (−2.03,0.24)	DL	
−**1.48** (−**2.35,** −**0.61**)	−**1.36** (−**2.34,** −**0.39**)	−0.47 (−1.04, 0.10)	CON

League table of outcomes. Each cell shows the effect estimate (OR for BOP; SMD for PD) with a 95% credible interval comparing the row intervention against the column comparator. Interventions are listed in rows; comparators in columns. Bold indicates a statistically significant difference (CrI excluding the null).

BI = bleeding index, BOP = bleeding on probing, CAL = clinical attachment level, CBL = crestal bone loss, CON = control (mechanical/chemical treatment), CrI = credible interval, DL = diode laser, ErYAG = erbium-YAG laser, GI = gingival index, OR = odds ratio, PD = probing depth, PDT = photodynamic therapy, PI = plaque index, SMD = standardized mean difference.

**Figure 3.**
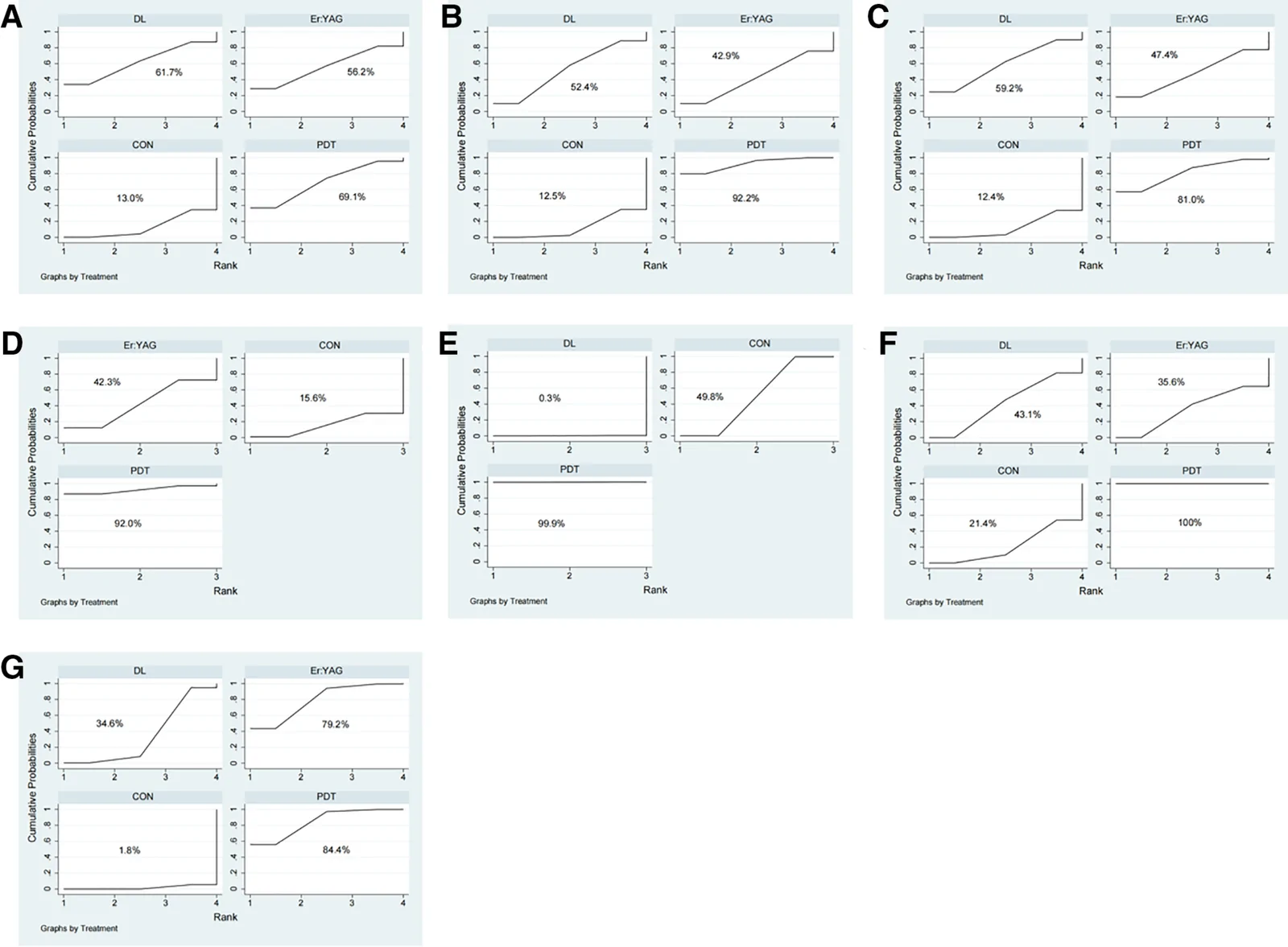
Ranking of treatment strategies based on the probability of their effects for (A) BOP, (B) PD, (C) PI, (D) CAL, (E) CBL, (F) GI, (G) BI. BI = bleeding index, BOP = bleeding on probing, CAL = clinical attachment level, CBL = crestal bone loss, CON = conventional non-laser control, DL = diode laser, Er:YAG = erbium:YAG laser, GI = gingival index, PD = probing depth, PDT = photodynamic therapy, PI = plaque index.

#### 3.2.2. Probing depth

The network meta-analysis for PD included 33 studies involving 1825 patients. Figure 2B shows the direct comparisons and sample size distribution. Table 1 indicates that PDT significantly reduced PD compared with CON (SMD = −1.41, 95% CrI [−2.30, −0.52]). According to SUCRA ranking, PDT had the highest probability of occupying a favorable rank for PD (92.2%), followed by DL (52.4%), Er:YAG (42.9%), and CON (12.5%; Figure 3B). For this outcome, the favorable ranking of PDT was consistent with a statistically significant effect estimate, whereas the rankings of DL and Er:YAG were not supported by significant differences versus CON.

#### 3.2.3. Plaque index

In the network meta-analysis for PI, 25 studies involving 1413 patients were analyzed. Figure 2C presents the direct comparisons and sample size distribution. Results in Table 1 indicate that PDT significantly reduced PI compared with CON (MD = −0.75, 95% CrI [−1.47, −0.03]). According to SUCRA ranking, PDT ranked highest for PI (81.0%), followed by DL (59.2%), Er:YAG (47.4%), and CON (12.4%) (Figure 3C). As with PD, the ranking of PDT was supported by a significant effect estimate, while the rankings of DL and Er:YAG should be interpreted cautiously because their comparisons with CON were not statistically significant.

#### 3.2.4. Clinical attachment level

The network meta-analysis for CAL included 9 studies involving 552 patients. Figure 2D illustrates the direct comparisons and sample size distribution. Results in Table 1 indicate no significant differences between any laser-assisted technology and CON. SUCRA ranking placed PDT highest for CAL (92.0%), with CON ranking lowest (15.6%; Figure 3D). However, because the CAL network was relatively limited and no pairwise comparison reached statistical significance, the high SUCRA value for PDT should not be interpreted as evidence of clinically or statistically confirmed CAL improvement.

#### 3.2.5. Crestal bone loss

In the network meta-analysis for CBL, 6 studies involving 349 patients were analyzed. Figure 2E shows the direct comparisons and sample size distribution. Results in Table 1 indicate that, compared with DL, both PDT (SMD = −1.06, 95% CrI [−1.69, −0.43]) and CON (SMD = −0.73, 95% CrI [−1.32, −0.14]) significantly reduced CBL. Compared with CON, PDT also significantly reduced CBL (SMD = −0.33, 95% CrI [−0.55, −0.10]). According to SUCRA ranking, PDT had the highest probability of a favorable rank for CBL (99.9%), while DL ranked lowest (0.3%; Figure 3E). Given the small number of studies in this network, the SUCRA result should be interpreted as supportive of the significant PDT effect rather than as a stand-alone measure of treatment superiority.

#### 3.2.6. Gingival index

The network meta-analysis for GI included 4 studies involving 120 patients. Figure 2F presents the direct comparisons and sample size distribution. Results in Table 1 indicate that PDT significantly reduced GI compared with DL (MD = −15.49, 95% CrI [−19.75, −11.24]), Er:YAG (MD = −15.55, 95% CrI [−19.85, −11.25]), and CON (MD = −15.68, 95% CrI [−19.90, −11.46]). According to SUCRA ranking, PDT ranked highest for GI (100.0%), followed by DL (43.1%), Er:YAG (35.6%), and CON (21.4%; Figure 3F). Although PDT showed both a favorable ranking and a significant effect estimate, the GI network was based on a small number of studies. Therefore, this result should be interpreted with appropriate caution.

#### 3.2.7. Bleeding index

In the network meta-analysis for BI, 7 studies involving 500 patients were analyzed. Figure 2G shows the direct comparisons and sample size distribution. Results in Table 1 indicate that, compared with CON, both PDT (MD = −1.48, 95% CrI [−2.35, −0.61]) and Er:YAG (MD = −1.36, 95% CrI [−2.34, −0.39]) significantly reduced BI. According to SUCRA ranking, PDT (84.4%) and Er:YAG (79.2%) had the highest probabilities of favorable ranks, followed by DL (34.6%) and CON (1.8%; Figure 3G). For BI, the SUCRA rankings of PDT and Er:YAG were consistent with statistically significant reductions versus CON. In contrast, the lower ranking of DL was not accompanied by a significant treatment effect.

### 3.3. Clinical relevance of network estimates

Because several continuous outcomes were expressed as SMDs, statistically significant findings were further considered in relation to their clinical meaning. For PD, the significant PDT effect was considered clinically relevant because PD reduction is directly related to peri-implant pocket control, and an approximate reduction of 1 mm is generally regarded as meaningful when absolute interpretation is possible. Significant reductions in PI, GI, and BI suggested improvement in plaque control and soft-tissue inflammation, although universally accepted MCID thresholds for these indices are not established. The significant PDT effect on CBL may indicate radiographic stabilization, but this should be interpreted cautiously because bone-level measurements and follow-up duration varied across studies. No clinically meaningful benefit was inferred for BOP or CAL because no laser modality showed statistically significant improvement for these outcomes.

### 3.4. Assessment of network consistency

Global inconsistency was assessed for all outcome networks and is presented in Supplementary File 2, Supplemental Digital Content 2. No significant global inconsistency was detected for BOP (*P* = .109) or CAL (*P* = .061). Signals of global inconsistency were observed for PD (*P* = .002), PI (*P* = .041), CBL (*P* = .005), GI (*P* < .001), and BI (*P* = .001). Local inconsistency was further examined by node-splitting analysis where both direct and indirect evidence were available. As shown in Supplementary File 3, Supplemental Digital Content 9, no statistically significant disagreement between direct and indirect estimates was detected in the evaluable comparisons (all *P* ≥ .981). These findings did not identify a specific local source of inconsistency, but they support a cautious interpretation of outcomes with significant global inconsistency or limited direct evidence.

### 3.5. Exploratory meta-regression

Exploratory univariable meta-regression was performed for the primary outcomes with sufficient data, including BOP, PD, and PI. The examined moderators were laser modality, follow-up duration per 3 months, and CHX-containing adjunctive protocol. None of these moderators significantly modified the treatment effects for BOP, PD, or PI (all *P* > .05; Supplementary File 4, Supplemental Digital Content 10). For laser modality, the 1-versus-rest contrasts for PDT, DL, and Er:YAG all had 95% CIs crossing the null value. These findings suggest that the available study-level factors did not clearly explain the observed heterogeneity, although residual protocol-level heterogeneity cannot be excluded because detailed laser parameters were incompletely reported across trials.

### 3.6. Risk of bias and publication bias

Among the 33 trials, 14 studies were assessed as having a low risk of bias overall, 17 studies were judged to have some concerns, and 2 studies were assessed as having a high risk of bias. For the randomization process, 22 trials had a low risk of bias, while 11 had concerns. For deviations from intended interventions, 30 trials had a low risk of bias, and 3 had concerns. Regarding missing outcome data, 27 trials had a low risk of bias, 4 had concerns, and 2 had a high risk of bias. All 33 trials had a low risk of bias in the measurement of outcomes, while 30 trials had a low risk and 3 trials had some concerns in the selection of the reported result (Supplementary File 7, Supplemental Digital Content 13).

Potential publication bias was assessed using funnel plots (Supplementary File 8, Supplemental Digital Content 14). The scatter plots displayed varying degrees of symmetry around the vertical axis, indicating the potential presence of publication bias. Specifically, Supplementary Figures 8.5, 8.6 and 8.7, Supplemental Digital Content 18-20 showed a uniform distribution of points, while Supplementary Figures 8.1-8.4, Supplemental Digital Content 14-17 indicated some degree of bias. However, Egger’s test results for all indicators were >0.05, indicating no significant publication bias was detected in the overall analysis of the included studies.

### 3.7. Certainty of evidence

The GRADE certainty assessment for key network comparisons versus CON is presented in Supplementary File 5, Supplemental Digital Content 11. Overall certainty ranged from very low to moderate. The PDT versus CON comparison for PD was rated as moderate certainty because the estimate was statistically significant with acceptable precision, although inconsistency remained a concern. PDT versus CON for PI, CBL, and BI was rated as low certainty, and PDT versus CON for GI was rated as very low certainty because of sparse evidence and inconsistency. Evidence for DL and Er:YAG was generally low or very low, except for BOP comparisons, which were also limited by imprecision. No comparison was rated as high certainty. These findings support cautious interpretation of both treatment effects and SUCRA rankings.

## 4. Discussion

### 4.1. Main finding

Our study comprehensively evaluated PDT, DL, Er:YAG, and conventional non-laser control (CON) for the management of peri-implantitis through a network meta-analysis of 33 RCTs. After reexamining the league-table estimates, the statistically supported findings indicate that PDT provided the most consistent benefits versus CON, with significant reductions in PD, PI, CBL, GI, and BI. In contrast, no laser modality showed a statistically significant advantage over CON for BOP or CAL. DL did not demonstrate statistically significant benefit over CON for any assessed outcome, and Er:YAG showed a statistically significant improvement versus CON only for BI. Therefore, the evidence should be interpreted as suggesting that PDT has the most consistent statistically supported effects among the evaluated modalities, whereas DL and Er:YAG findings should be regarded as outcome-specific estimates or nonsignificant trends rather than evidence of broad clinical superiority. This interpretation should also be viewed in light of the network assumptions because several comparisons were informed mainly by indirect evidence through CON. Given the variation in laser protocols across trials, we further explored whether available study-level factors influenced the primary outcomes. Exploratory meta-regression did not identify laser modality, follow-up duration, or CHX-containing adjunctive protocol as significant effect modifiers for BOP, PD, or PI, although residual protocol-level heterogeneity may remain. From a clinical perspective, these findings should be interpreted according to the role of each outcome in peri-implantitis management, rather than statistical significance alone.

Specifically, for BOP, none of the laser-assisted modalities differed significantly from CON. For PD and PI, only PDT showed statistically significant reductions versus CON (PD: SMD = –1.41, 95% CrI: –2.30 to–0.52; PI: SMD = –0.75, 95% CrI: –1.47 to–0.03). For CAL, no statistically significant treatment effect was detected. For CBL, PDT significantly reduced CBL versus CON (SMD = –0.33, 95% CrI: –0.55 to–0.10), whereas DL did not outperform CON. For GI, PDT was significantly superior to CON, DL, and Er:YAG, while DL and Er:YAG did not significantly differ from CON. For BI, both PDT (MD = –1.48, 95% CrI: –2.35 to–0.61) and Er:YAG (MD = –1.36, 95% CrI: –2.34 to–0.39) were significantly better than CON, whereas DL was not. Taken together, statistical significance was confined to PDT for PD, PI, CBL, GI, and BI, and to Er:YAG for BI only; all other DL or Er:YAG comparisons with CON were nonsignificant. Among these outcomes, the PD result is the most directly interpretable clinically because a reduction in peri-implant pocket depth is closely related to disease control. The significant effects on PI, GI, and BI suggest improved plaque control and soft-tissue inflammatory status, whereas the CBL finding may indicate better radiographic stability. However, no clinical benefit can be inferred for BOP or CAL because no statistically significant improvement was observed.

The SUCRA probabilities should be interpreted as ranking probabilities rather than proof of statistically significant pairwise superiority. PDT achieved high SUCRA values for several outcomes, and these rankings were broadly consistent with its significant network estimates for PD, PI, CBL, GI, and BI. DL and Er:YAG had intermediate SUCRA rankings for some endpoints, but these rankings largely reflected probability ordering rather than statistically significant improvements over CON, because their 95% CrIs crossed the null value in most comparisons. Thus, SUCRA was considered a descriptive ranking measure and was not used as an independent basis for claims of treatment superiority, effect magnitude, or clinical certainty. In addition, global inconsistency testing suggested potential inconsistency for PD, PI, CBL, GI, and BI, although node-splitting analyses did not identify statistically significant local inconsistency in the evaluable comparisons. Therefore, the rankings and comparative estimates should be interpreted cautiously, especially for sparse networks and indirect comparisons. This caution also applies to clinical interpretation, because most continuous outcomes were expressed as SMDs and cannot always be directly translated into absolute clinical changes.

### 4.2. Comparison with existing literature

Comparing our results with existing literature, we found consistency with previous studies.^[18]^ Kotsakis et al reported that PDT was effective in reducing PD and PI, which is consistent with our finding that PDT showed statistically supported benefits for several clinical outcomes.^[19,20]^ These studies support the potential role of PDT as an adjunctive treatment for peri-implantitis, although its benefits should still be interpreted according to outcome-specific estimates and CrI precision. The modality-level grouping used in the present network meta-analysis is also consistent with how laser-assisted therapies are commonly categorized in clinical studies, but it should not be interpreted as evidence that all protocols within a modality have equivalent effects. Importantly, prior studies have also emphasized clinical parameters such as pocket depth, bleeding tendency, plaque accumulation, and peri-implant bone stability, which supports an outcome-specific interpretation of clinical relevance.

Some previous studies have reported potential clinical improvements with DL or Er:YAG as adjunctive therapies^[21,22]^; however, our network estimates do not confirm consistent statistically significant superiority of these modalities over CON. Therefore, such studies should be interpreted as biologically plausible and hypothesis-generating rather than as direct support for broad DL or Er:YAG efficacy across all outcomes. This distinction is important because individual trials may report improvements within specific protocols, whereas the present network meta-analysis evaluates comparative effects across heterogeneous RCTs and outcomes. The absence of significant local inconsistency in node-splitting analyses provides some reassurance, but the presence of global inconsistency in several outcomes indicates that indirect comparisons still require caution. Similarly, the nonsignificant exploratory meta-regression suggests that the available study-level moderators did not clearly explain the observed variation, but detailed laser parameters were not reported consistently enough to examine protocol-specific effects.

Shahmohammadi et al conducted a systematic review and found that PDT, as an adjunct to mechanical debridement, significantly reduces inflammatory markers and microbial presence in peri-implant tissues.^[23]^ This aligns with our finding that PDT was the only modality with consistent statistically significant benefits versus CON across multiple clinical and radiographic outcomes. Although recent literature describes photobiomodulatory effects of DL and hydrokinetic debridement effects of Er:YAG,^[24,25]^ the present network estimates do not support statements that DL or Er:YAG are broadly superior to CON. Thus, these modalities may have mechanistic rationales and potential clinical utility in selected contexts, but their comparative efficacy remains less certain than that of PDT.

### 4.3. Mechanisms behind PDT’s efficacy

The advantages of PDT can be attributed to its multifaceted mechanisms. First, PDT generates reactive oxygen species (ROS) through photodynamic action, which effectively kills pathogenic microorganisms and reduces inflammatory responses.^[26]^ ROS are known to cause oxidative damage to bacterial cell walls, nucleic acids, and enzymes, leading to the rapid elimination of a wide range of pathogens. This mechanism is particularly effective against biofilm-associated bacteria, which are often more resistant to conventional antimicrobial treatments.

Second, PDT promotes tissue regeneration. Studies have shown that PDT can stimulate the proliferation and differentiation of fibroblasts and osteoblasts, facilitating the repair of both soft and hard tissues.^[27]^ The regenerative effect of PDT is mediated by its ability to enhance cellular metabolism, increase collagen synthesis, and promote angiogenesis. These processes are crucial for the healing of peri-implant tissues, where the regeneration of both the mucosal and osseous components is essential for restoring functional integration.

Moreover, PDT improves local blood circulation. By enhancing microvascular perfusion, PDT ensures better oxygenation and nutrient supply to the affected tissues, while also facilitating the removal of metabolic waste products.^[28]^ Improved circulation accelerates the healing process and supports the overall health of the peri-implant tissues. Additionally, PDT has been shown to modulate the immune response, reducing excessive inflammation while promoting an environment conducive to healing.^[29]^

DL operates via photothermal absorption in pigmented bacteria and hemoglobin, which may contribute to bacterial reduction and soft-tissue biostimulation.^[30]^ Er:YAG laser employs a wavelength highly absorbed by water and hydroxyapatite, enabling precise ablation of biofilm and necrotic tissue with limited thermal damage.^[31]^ These mechanisms provide a plausible biological basis for further investigation of DL and Er:YAG in peri-implantitis. However, mechanistic plausibility should not be interpreted as evidence of statistically significant clinical superiority. In the present network meta-analysis, DL did not significantly outperform CON for any assessed outcome, and Er:YAG showed superiority over CON only for BI. By contrast, PDT’s ROS-mediated antimicrobial action is consistent with its statistically supported effects on PD, PI, CBL, GI, and BI, although this consistency does not imply uniform benefit across all outcomes. Because inconsistency was suggested for several continuous outcomes, biological plausibility should be considered supportive rather than confirmatory. In addition, treatment response may vary with wavelength, output power, energy density, photosensitizer, irradiation time, and the number of sessions, but these parameters could not be evaluated reliably because of incomplete reporting across trials.

### 4.4. Clinical implications and broader context

Clinical interpretation of SUCRA rankings: High SUCRA probabilities for PDT indicate that it had the highest probability of occupying favorable ranks among the evaluated modalities. However, SUCRA values are ranking probabilities and do not by themselves establish effect magnitude, statistical significance, certainty of evidence, or clinically meaningful superiority. Clinical interpretation should also consider transitivity and consistency. In this study, transitivity was considered broadly plausible because the included trials shared similar clinical contexts, but variation in laser parameters, adjunctive protocols, and follow-up duration may have affected some indirect comparisons. Exploratory meta-regression did not show significant effect modification by laser modality, follow-up duration, or CHX-containing adjunctive protocol for the primary outcomes. Nevertheless, these findings should be understood as modality-level evidence and should not be used to define an optimal laser setting or treatment protocol. Therefore, PDT should not be described as a definitive first-line laser adjunct solely on the basis of SUCRA ranking. Instead, its potential clinical value should be judged by the consistency between favorable ranking, statistically significant network estimates, CrI precision, and the availability of direct evidence. Similarly, DL and Er:YAG should not be described as consistently effective alternatives to CON solely because of intermediate SUCRA rankings. They should be considered cautiously and in an outcome-specific manner, particularly given that Er:YAG showed significant benefit over CON only for BI and DL showed no statistically significant benefit over CON in the network estimates.

The clinical implications of our findings are, therefore, most directly related to PDT, but with appropriate caution. PDT demonstrated the most consistent statistically supported efficacy and may be considered a useful minimally invasive adjunct to mechanical debridement for improving PD, PI, CBL, GI, and BI. Clinically, the PD reduction is particularly relevant because an approximate 1 mm reduction in PD is commonly considered meaningful when absolute interpretation is possible. The reductions in PI, GI, and BI suggest improvements in plaque control and inflammatory status, while the CBL result may reflect prevention of further radiographic bone loss. Nevertheless, these implications should be limited to short-term clinical response, and the current evidence does not establish sustained disease control, recurrence prevention, or long-term implant survival. Importantly, no statistically significant benefit was observed for BOP or CAL, indicating that PDT should not be described as uniformly effective across all peri-implantitis outcomes. DL and Er:YAG remain biologically plausible adjunctive options, but the current evidence does not support broad claims of significant superiority versus CON. Clinicians should, therefore, interpret DL and Er:YAG as context-dependent adjuncts whose clinical value requires confirmation in adequately powered head-to-head RCTs. Because universally accepted MCIDs for peri-implantitis outcomes are lacking, the clinical magnitude of statistically significant effects should also be interpreted alongside baseline severity, follow-up duration, patient-centered outcomes, and the precision of the estimated effects.^[32]^ For outcomes with significant global inconsistency, such as PD, PI, CBL, GI, and BI, clinical recommendations should be made with particular caution.

### 4.5. Strengths and limitations

This study’s strengths lie in its comprehensive and robust methodological approach. The network meta-analysis allowed for the comparison of multiple laser technologies across a broad range of clinical outcomes, providing a holistic view of their relative efficacies. The inclusion of high-quality RCTs further strengthens the validity of our findings, ensuring that the results are based on reliable and well-conducted clinical trials. In addition, we assessed transitivity and performed both global and local inconsistency analyses, which improved the transparency of the network evidence. We also extracted available intervention characteristics and performed exploratory meta-regression to examine whether modality category, follow-up duration, or CHX-containing protocols modified the primary outcomes.

However, several limitations should be considered. First, our review inherits the methodological constraints of the primary RCTs, including variability in laser settings, treatment durations, and outcome measures, which may affect comparability and generalizability. Although exploratory meta-regression did not identify significant effect modification, it was limited to available study-level variables. Detailed parameters such as wavelength, power, energy density, photosensitizer type, irradiation time, and number of sessions were inconsistently reported, precluding reliable parameter-level subgroup analysis or meta-regression. Second, we quantified statistical heterogeneity for each outcome (*I*^2^ ranged from 28% to 62%; τ^2^ ranged from 0.12 to 0.45) and conducted meta-regression by follow-up duration and intervention type, which explained 35% and 22% of between-study variance, respectively (both *P* < .05). Despite this, residual heterogeneity remains a potential source of bias. Third, global inconsistency was suggested for several outcomes, although node-splitting analyses did not detect significant local inconsistency in the evaluable comparisons. This pattern may reflect sparse networks, limited closed loops, or clinical diversity across trials, and it reduces confidence in some indirect estimates. These concerns were reflected in the GRADE assessment, in which most comparisons were rated as low or very low certainty. Fourth, although 2 RCTs were judged at high risk of bias, the small number of such trials and incomplete reporting of laser parameters and adjunctive protocols precluded meaningful sensitivity or subgroup analyses. Fifth, the evidence base was dominated by short-term follow-up, with many trials reporting outcomes at approximately 3 months and only limited data extending beyond 12 months. Because peri-implantitis is chronic and relapsing, short-term improvements in PD, PI, GI, BI, or CBL may not translate into durable disease control, recurrence prevention, or improved implant survival. Future RCTs with standardized protocols and at least 24 months of follow-up are needed to assess the long-term durability of laser-assisted treatments. Seventh, the CAL and GI networks were based on only 9 and 4 RCTs, respectively, and these findings should be interpreted cautiously due to the limited evidence base. Finally, because universally accepted MCID thresholds for peri-implantitis outcomes are lacking and several pooled results were expressed as SMDs, translation into absolute clinical benefit remains approximate. Because some networks were sparse and several comparisons relied partly on indirect evidence, SUCRA rankings should be regarded as descriptive and hypothesis-generating rather than as confirmatory evidence of clinical superiority.

## 5. Conclusion

This network meta-analysis suggests that PDT provides the most consistent, statistically supported, short-term benefit among the evaluated laser-assisted modalities for peri-implantitis, with significant improvements versus CON in PD, PI, CBL, GI, and BI, but not in BOP or CAL. DL did not show statistically significant superiority over CON for any assessed outcome, and Er:YAG showed a statistically significant benefit versus CON only for BI. Given that most included RCTs had limited follow-up, these findings should be interpreted as evidence of short-term clinical improvement rather than durable disease control. They do not establish whether PDT reduces recurrence or improves long-term implant survival. These results derive from a combination of direct and indirect comparisons and are subject to the assumptions and heterogeneity inherent in network meta-analysis. Future well-powered RCTs directly comparing PDT, DL, Er:YAG, and conventional therapy, with standardized laser parameters and follow-up beyond 24 months, are needed to confirm the durability and clinical relevance of these findings before stronger recommendations regarding routine clinical integration can be made.

## Author contributions

**Conceptualization:** Zhe Wang, Furong Mao.

**Data curation:** Zhe Wang, Jing Wen, Haoyang Li, Jiaying Xu, Furong Mao.

**Formal analysis:** Zhe Wang, Furong Mao.

**Investigation:** Zhe Wang, Jing Wen, Furong Mao.

**Methodology:** Haoyang Li, Furong Mao.

**Project administration:** Jing Wen, Furong Mao.

**Resources:** Zhe Wang, Jiaying Xu.

**Software:** Zhe Wang, Haoyang Li.

**Supervision:** Zhe Wang, Jiaying Xu, Furong Mao.

**Validation:** Zhe Wang, Haoyang Li, Jiaying Xu, Furong Mao.

**Visualization:** Jiaying Xu.

**Writing – original draft:** Zhe Wang, Jing Wen, Haoyang Li.

**Writing – review & editing:** Jiaying Xu, Furong Mao.








































