# Supplementary material for: Comparative effectiveness of novel laser-assisted technologies for treating peri-implantitis: A network meta-analysis of randomized controlled trials
Source: Medicine (Baltimore). 2026 Oct 30;105(44):e51256. doi: 10.1097/MD.0000000000051256 (PMC13727279; doi:10.1097/MD.0000000000051256)
Supplement: Supplementary file 1 [file medi-105-e51256-s001.docx]

Supplementary 1: Search Strategy

***Search Strategy (PubMed):***

| #8 | Search: ((((((((((Peri-Implantitis[MeSH Terms]) OR (Peri-Implantitis[Title/Abstract])) OR (Periimplantitis[Title/Abstract])) OR ("Peri-implant Disease"[Title/Abstract])) OR ("Peri-implant Infection"[Title/Abstract])) OR (Implantitis[Title/Abstract])) OR ("Peri-implant Bone Loss"[Title/Abstract])) OR ("Peri-implant Mucositis"[Title/Abstract])) OR ("Peri-implant Inflammatory Disease"[Title/Abstract])) AND ((((((((((((((Laser Therapy[MeSH Terms]) OR ("Laser Therapy"[Title/Abstract])) OR ("Laser Treatment"[Title/Abstract])) OR (Phototherapy[Title/Abstract])) OR ("Laser-Assisted Therapy"[Title/Abstract])) OR ("Diode Laser"[Title/Abstract])) OR ("Er:YAG Laser"[Title/Abstract])) OR ("Nd:YAG Laser"[Title/Abstract])) OR ("CO2 Laser"[Title/Abstract])) OR ("Low-Level Laser Therapy"[Title/Abstract])) OR ("High-Power Laser Therapy"[Title/Abstract])) OR ("Laser Decontamination"[Title/Abstract])) OR ("Laser Ablation"[Title/Abstract])) OR ("Laser Irradiation"[Title/Abstract]))) AND ((randomized controlled trial[pt] OR controlled clinical trial[pt] OR randomized[tiab] OR placebo[tiab] OR drug therapy[sh] OR randomly[tiab] OR trial[tiab] OR groups[tiab]) NOT (animals[mh] NOT humans[mh])) |
| --- | --- |
| #7 | Search: (randomized controlled trial[pt] OR controlled clinical trial[pt] OR randomized[tiab] OR placebo[tiab] OR drug therapy[sh] OR randomly[tiab] OR trial[tiab] OR groups[tiab]) NOT (animals[mh] NOT humans[mh]) |
| #6 | Search: (((((((((((((Laser Therapy[MeSH Terms]) OR ("Laser Therapy"[Title/Abstract])) OR ("Laser Treatment"[Title/Abstract])) OR (Phototherapy[Title/Abstract])) OR ("Laser-Assisted Therapy"[Title/Abstract])) OR ("Diode Laser"[Title/Abstract])) OR ("Er:YAG Laser"[Title/Abstract])) OR ("Nd:YAG Laser"[Title/Abstract])) OR ("CO2 Laser"[Title/Abstract])) OR ("Low-Level Laser Therapy"[Title/Abstract])) OR ("High-Power Laser Therapy"[Title/Abstract])) OR ("Laser Decontamination"[Title/Abstract])) OR ("Laser Ablation"[Title/Abstract])) OR ("Laser Irradiation"[Title/Abstract]) |
| #5 | Search: (((((((((((("Laser Therapy"[Title/Abstract])) OR ("Laser Treatment"[Title/Abstract])) OR (Phototherapy[Title/Abstract])) OR ("Laser-Assisted Therapy"[Title/Abstract])) OR ("Diode Laser"[Title/Abstract])) OR ("Er:YAG Laser"[Title/Abstract])) OR ("Nd:YAG Laser"[Title/Abstract])) OR ("CO2 Laser"[Title/Abstract])) OR ("Low-Level Laser Therapy"[Title/Abstract])) OR ("High-Power Laser Therapy"[Title/Abstract])) OR ("Laser Decontamination"[Title/Abstract])) OR ("Laser Ablation"[Title/Abstract])) OR ("Laser Irradiation"[Title/Abstract]) |
| #4 | Search: (Laser Therapy[MeSH Terms]) |
| #3 | Search: ((((((((Peri-Implantitis[MeSH Terms]) OR (Peri-Implantitis[Title/Abstract])) OR (Periimplantitis[Title/Abstract])) OR ("Peri-implant Disease"[Title/Abstract])) OR ("Peri-implant Infection"[Title/Abstract])) OR (Implantitis[Title/Abstract])) OR ("Peri-implant Bone Loss"[Title/Abstract])) OR ("Peri-implant Mucositis"[Title/Abstract])) OR ("Peri-implant Inflammatory Disease"[Title/Abstract]) |
| #2 | Search: (((((((Peri-Implantitis[Title/Abstract])) OR (Periimplantitis[Title/Abstract])) OR ("Peri-implant Disease"[Title/Abstract])) OR ("Peri-implant Infection"[Title/Abstract])) OR (Implantitis[Title/Abstract])) OR ("Peri-implant Bone Loss"[Title/Abstract])) OR ("Peri-implant Mucositis"[Title/Abstract])) OR ("Peri-implant Inflammatory Disease"[Title/Abstract]) |
| #1 | Search: (Peri-Implantitis[MeSH Terms]) |

***Search Strategy (Ovid MEDLINE):***

1. exp Peri-implantitis/

2. (Peri-implantitis or Periimplantitis or “Peri-implant Disease” or “Peri-implant Infection” or Implantitis or “Peri-implant Bone Loss” or “Peri-implant Mucositis” or “Peri-implant Inflammatory Disease”).ti,ab.

3. 1 or 2

4. exp Laser Therapy/

5. (Laser Therapy or Laser Treatment or Phototherapy or “Laser-Assisted Therapy” or “Diode Laser” or “Er:YAG Laser” or “Nd:YAG Laser” or “CO2 Laser” or “Low-Level Laser Therapy” or “High-Power Laser Therapy” or “Laser Decontamination” or “Laser Ablation” or “Laser Irradiation”).ti,ab.

6. 4 or 5

7. randomized controlled trial.pt. or controlled clinical trial.pt.

8. (randomized or placebo or randomly or trial or groups).ti,ab.

9. drug therapy.sh.

10. 7 or 8 or 9

11. exp Animals/ not Humans/

12. 10 not 11

13. 3 and 6 and 12

***Search Strategy (Ovid MEDLINE):***

1. 'peri-implantitis'/exp

2. (peri-implantitis OR periimplantitis OR 'peri-implant disease' OR 'peri-implant infection' OR implantitis OR 'peri-implant bone loss' OR 'peri-implant mucositis' OR 'peri-implant inflammatory disease').ti,ab,kw.

3. 1 OR 2

4. 'laser therapy'/exp

5. (laser therapy OR laser treatment OR phototherapy OR 'laser-assisted therapy' OR 'diode laser' OR 'Er:YAG laser' OR 'Nd:YAG laser' OR 'CO2 laser' OR 'low-level laser therapy' OR 'high-power laser therapy' OR 'laser decontamination' OR 'laser ablation' OR 'laser irradiation').ti,ab,kw.

6. 4 OR 5

7. 'randomized controlled trial'/de OR 'controlled clinical trial'/de

8. (randomized OR placebo OR randomly OR trial OR groups).ti,ab.

9. 7 OR 8

10. animal/lim NOT human/lim

11. 9 NOT 10

12. 3 AND 6 AND 11

***Search Strategy (Web of Science):***

1. TS=(peri-implantitis)

2. TS=(periimplantitis OR “peri-implant disease” OR “peri-implant infection” OR implantitis OR “peri-implant bone loss” OR “peri-implant mucositis” OR “peri-implant inflammatory disease”)

3. #1 OR #2

4. TS=(“laser therapy”)

5. TS=(“laser treatment” OR phototherapy OR “laser-assisted therapy” OR “diode laser” OR “Er:YAG laser” OR “Nd:YAG laser” OR “CO2 laser” OR “low-level laser therapy” OR “high-power laser therapy” OR “laser decontamination” OR “laser ablation” OR “laser irradiation”)

6. #4 OR #5

7. TS=(randomized OR randomised OR placebo OR randomly OR trial OR groups)

8. TS=(animal*)

9. TS=(human*)

10. #7 NOT (#8 NOT #9)

11. #3 AND #6 AND #10
