# Supplementary material for: Comparative effectiveness of novel laser-assisted technologies for treating peri-implantitis: A network meta-analysis of randomized controlled trials
Source: Medicine (Baltimore). 2026 Oct 30;105(44):e51256. doi: 10.1097/MD.0000000000051256 (PMC13727279; doi:10.1097/MD.0000000000051256)
Supplement: Supplementary file 2 [file medi-105-e51256-s009.docx]

# Supplementary 3: Local inconsistency analyses

3.1 Local Inconsistency Assessment for PD

| **Side** | | **Direct** | | **Indirect** | | **Difference** | | **P>\|z\|** | **tau** |
| --- | --- | --- | --- | --- | --- | --- | --- | --- | --- |
|  |  | **Coef.** | **Std. Err.** | **Coef.** | **Std. Err.** | **Coef.** | **Std. Err.** |  |  |
| DL | MD | 0.595 | 0.519 | 0.559 | 67.397 | 0.036 | 67.399 | 1.000 | 1.686 |
| Er:YAG | MD | 0.432 | 0.653 | 1.186 | 239.089 | -0.753 | 239.090 | 0.997 | 1.686 |
| MD | PDT | -1.408 | 0.454 | -1.161 | 163.333 | -0.246 | 163.334 | 0.999 | 1.686 |

3.2 Local Inconsistency Assessment for PI

| **Side** | | **Direct** | | **Indirect** | | **Difference** | | **P>\|z\|** | **tau** |
| --- | --- | --- | --- | --- | --- | --- | --- | --- | --- |
|  |  | **Coef.** | **Std. Err.** | **Coef.** | **Std. Err.** | **Coef.** | **Std. Err.** |  |  |
| DL | MD | 0.478 | 0.403 | 0.300 | 79.086 | 0.178 | 79.087 | 0.998 | 1.094 |
| Er:YAG | MD | 0.339 | 0.475 | 0.970 | 258.285 | -0.631 | 258.286 | 0.998 | 1.094 |
| MD | PDT | -0.752 | 0.368 | -0.945 | 200.061 | 0.193 | 200.062 | 0.999 | 1.094 |

3.3 Local Inconsistency Assessment for CBL

| **Side** | | **Direct** | | **Indirect** | | **Difference** | | **P>\|z\|** | **tau** |
| --- | --- | --- | --- | --- | --- | --- | --- | --- | --- |
|  |  | **Coef.** | **Std. Err.** | **Coef.** | **Std. Err.** | **Coef.** | **Std. Err.** |  |  |
| DL | MD | -0.731 | 0.299 | 0.167 | 142.62 | -0.897 | 142.62 | 0.995 | 0 |
| MD | PDT | -0.326 | 0.116 | 1.466 | 283.82 | -1.792 | 283.82 | 0.995 | 0 |

3.4 Local Inconsistency Assessment for GI

| **Side** | | **Direct** | | **Indirect** | | **Difference** | | **P>\|z\|** | **tau** |
| --- | --- | --- | --- | --- | --- | --- | --- | --- | --- |
|  |  | **Coef.** | **Std. Err.** | **Coef.** | **Std. Err.** | **Coef.** | **Std. Err.** |  |  |
| DL | MD | 0.184 | 0.273 | 3.949 | 224.908 | -3.766 224.908 | 0.987 | 0.086 | 0.184 |
| Er:YAG | MD | 0.125 | 0.418 | 0.369 | 631.674 | -0.245 631.674 | 1 | 0.086 | 0.125 |
| MD | PDT | -15.678 | 2.154 | -0.367 | 632.47 | -15.311 632.473 | 0.981 | 0.086 | -15.678 |

3.5 Local Inconsistency Assessment for BI

| **Side** | | **Direct** | | **Indirect** | | **Difference** | | **P>\|z\|** | **tau** |
| --- | --- | --- | --- | --- | --- | --- | --- | --- | --- |
|  |  | **Coef.** | **Std. Err.** | **Coef.** | **Std. Err.** | **Coef.** | **Std. Err.** |  |  |
| DL | MD | 0.47 | 0.291 | 0.73 | 183.599 | -0.261 183.600 | 0.999 | 0.478 | 0.47 |
| Er:YAG | MD | 1.363 | 0.499 | 0.937 | 635.168 | 0.426 635.168 | 0.999 | 0.478 | 1.363 |
| MD | PDT | -1.478 | 0.443 | -0.903 | 448.126 | -0.575 448.126 | 0.999 | 0.478 | -1.478 |
