# Supplementary material for: Comparative effectiveness of novel laser-assisted technologies for treating peri-implantitis: A network meta-analysis of randomized controlled trials
Source: Medicine (Baltimore). 2026 Oct 30;105(44):e51256. doi: 10.1097/MD.0000000000051256 (PMC13727279; doi:10.1097/MD.0000000000051256)
Supplement: Supplementary file 3 [file medi-105-e51256-s010.docx]

# Supplementary 4: Exploratory univariable meta-regression for primary outcomes

| **Outcome** | **Comparisons, n** | **Moderator** | **β** | **95% CI** | **P value** |
| --- | --- | --- | --- | --- | --- |
| BOP | 24 | PDT vs non-PDT | -0.12 | -1.51 to 1.27 | 0.861 |
| BOP | 24 | DL vs non-DL | 0 | -1.59 to 1.60 | 0.998 |
| BOP | 24 | Er:YAG vs non-Er:YAG | 0.15 | -1.42 to 1.72 | 0.846 |
| BOP | 24 | Follow-up duration, per 3 months | -0.13 | -0.54 to 0.28 | 0.512 |
| BOP | 24 | CHX-containing adjunctive protocol | 0.45 | -0.94 to 1.85 | 0.506 |
| PD | 31 | PDT vs non-PDT | -0.82 | -2.04 to 0.39 | 0.176 |
| PD | 31 | DL vs non-DL | 0.27 | -1.05 to 1.60 | 0.674 |
| PD | 31 | Er:YAG vs non-Er:YAG | 0.93 | -0.62 to 2.47 | 0.23 |
| PD | 31 | Follow-up duration, per 3 months | 0.05 | -0.30 to 0.40 | 0.776 |
| PD | 31 | CHX-containing adjunctive protocol | -0.27 | -1.57 to 1.03 | 0.676 |
| PI | 22 | PDT vs non-PDT | -0.54 | -1.40 to 0.33 | 0.209 |
| PI | 22 | DL vs non-DL | 0.12 | -0.83 to 1.06 | 0.796 |
| PI | 22 | Er:YAG vs non-Er:YAG | 0.65 | -0.41 to 1.71 | 0.215 |
| PI | 22 | Follow-up duration, per 3 months | 0 | -0.22 to 0.23 | 0.976 |
| PI | 22 | CHX-containing adjunctive protocol | 0.09 | -0.81 to 0.99 | 0.837 |
