# Supplementary material for: Comparative effectiveness of novel laser-assisted technologies for treating peri-implantitis: A network meta-analysis of randomized controlled trials
Source: Medicine (Baltimore). 2026 Oct 30;105(44):e51256. doi: 10.1097/MD.0000000000051256 (PMC13727279; doi:10.1097/MD.0000000000051256)
Supplement: Supplementary file 4 [file medi-105-e51256-s011.docx]

# Supplementary 5: GRADE certainty for the network comparisons

| **Outcome** | **Main network comparison** | **Network estimate (OR/SMD, 95% CrI)** | **Risk of bias** | **Inconsistency** | **Indirectness** | **Imprecision** | **Publication bias** | **Overall certainty** |
| --- | --- | --- | --- | --- | --- | --- | --- | --- |
| BOP | PDT vs CON | OR 0.46 (0.18 to 1.19) | Some concerns | Not serious | Not serious | Serious | Undetected | Low |
| BOP | DL vs CON | OR 0.49 (0.14 to 1.78) | Some concerns | Not serious | Serious | Serious | Undetected | Low |
| BOP | Er:YAG vs CON | OR 0.54 (0.14 to 2.11) | Some concerns | Not serious | Serious | Serious | Undetected | Low |
| PD | PDT vs CON | SMD -1.41 (-2.30 to -0.52) | Some concerns | Serious | Not serious | Not serious | Undetected | Moderate |
| PD | DL vs CON | SMD -0.59 (-1.61 to 0.42) | Some concerns | Serious | Serious | Serious | Undetected | Very low |
| PD | Er:YAG vs CON | SMD -0.43 (-1.71 to 0.85) | Some concerns | Serious | Serious | Serious | Undetected | Very low |
| PI | PDT vs CON | SMD -0.75 (-1.47 to -0.03) | Some concerns | Serious | Not serious | Serious | Undetected | Low |
| PI | DL vs CON | SMD -0.48 (-1.27 to 0.31) | Some concerns | Serious | Serious | Serious | Undetected | Very low |
| PI | Er:YAG vs CON | SMD -0.34 (-1.27 to 0.59) | Some concerns | Serious | Serious | Serious | Undetected | Very low |
| CAL | PDT vs CON | SMD -0.59 (-1.20 to 0.03) | Some concerns | Not serious | Serious | Serious | Undetected | Low |
| CAL | Er:YAG vs CON | SMD -0.13 (-0.58 to 0.32) | Some concerns | Not serious | Serious | Serious | Undetected | Low |
| CBL | PDT vs CON | SMD -0.33 (-0.55 to -0.10) | Some concerns | Serious | Serious | Not serious | Undetected | Low |
| CBL | DL vs CON | SMD 0.73 (0.14 to 1.32) | Some concerns | Serious | Serious | Serious | Undetected | Very low |
| GI | PDT vs CON | SMD -15.68 (-19.90 to -11.46) | Some concerns | Serious | Serious | Serious | Undetected | Very low |
| GI | DL vs CON | SMD -0.18 (-0.72 to 0.35) | Some concerns | Serious | Serious | Serious | Undetected | Very low |
| GI | Er:YAG vs CON | SMD -0.12 (-0.94 to 0.69) | Some concerns | Serious | Serious | Serious | Undetected | Very low |
| BI | PDT vs CON | SMD -1.48 (-2.35 to -0.61) | Some concerns | Serious | Not serious | Serious | Undetected | Low |
| BI | Er:YAG vs CON | SMD -1.36 (-2.34 to -0.39) | Some concerns | Serious | Serious | Serious | Undetected | Very low |
| BI | DL vs CON | SMD -0.47 (-1.04 to 0.10) | Some concerns | Serious | Serious | Serious | Undetected | Very low |

Note: BOP, bleeding on probing; PD, probing depth; PI, plaque index; CAL, clinical attachment level; CBL, crestal bone loss; GI, gingival index; BI, bleeding index; PDT, photodynamic therapy; DL, diode laser; Er:YAG, erbium:YAG laser; CON, conventional non-laser control; OR, odds ratio; SMD, standardized mean difference; CrI, credible interval. For BOP, estimates are presented as ORs; for continuous outcomes, estimates are presented as SMDs. Randomized evidence was initially rated as high certainty and downgraded for concerns related to risk of bias, inconsistency, indirectness, imprecision, and publication bias. “Undetected” indicates that no clear evidence of publication bias was found based on funnel plot inspection and Egger’s test.
