# Supplementary material for: Comparative effectiveness of novel laser-assisted technologies for treating peri-implantitis: A network meta-analysis of randomized controlled trials
Source: Medicine (Baltimore). 2026 Oct 30;105(44):e51256. doi: 10.1097/MD.0000000000051256 (PMC13727279; doi:10.1097/MD.0000000000051256)
Supplement: Supplementary file 5 [file medi-105-e51256-s012.docx]

# Supplementary 6: Characteristics of studies and subjects included in the review

| **Study** | **Subjects**  **(intervention/ control)** | **Sex (male/female)**  **(intervention/ control)** | **Mean age**  **(intervention/ control)** | **Intervention detail** | | **Follow-up** | **Outcome** |
| --- | --- | --- | --- | --- | --- | --- | --- |
|  |  |  |  | **Intervention** | **Control** |  |  |
| Schwarz et al. (2005) | 20 (10/10) | 6/4 vs. 6/4 | 48 vs. 51 | Er:YAG | MD+CHX | 6 months | BOP, PD, PI, CAL |
| Schwarz et al. (2006) | 20 (10/10) | 4/6 vs. 5/5 | 56±14 vs. 52±11 | Er:YAG | MD | 12 months | BOP, PD, PI, CAL |
| Schwarz et al. (2011) | 31 (16/15) | 6/9 vs. 5/10 | 60.8±10.9 | Er:YAG | MD | 6 months | BOP, PD, PI, CAL |
| Schwarz et al. (2012) | 24 (10/14) | 3/7 vs. 5/9 | 62.3±10.0 vs. 62.3±10.0 | Er:YAG | MD+CHX | 24 months | BOP, PD, PI, CAL |
| Tenore et al. (2020) | 23 (11/12) | 7/4 vs. 7/5 | 55.73 vs. 56.42 | DL | MD | 3 months | BOP, PD |
| Ahmed et al. (2020) | 40 (20/20) | 20/0 vs. 20/0 | 48.9±4.5 vs. 50.7±5.9 | PDT | MD | 6 months | BOP, PD, PI, CBL |
| Arısan et al. (2015) | 48 (24/24) | 3/7 vs. 3/7 | 55.1±12.4 | DL | MD | 6 months | BOP, PD, PI, CBL |
| Wang et al. (2019) | 131 (65/66) | 23/42 vs. 25/41 | 44.1±9.8 vs. 42.6±13.0 | PDT | MD+CHX | 6 months | PD, CAL |
| Amri et al. (2016) | 67 (34/33) | 19/15 vs. 17/16 | 53.6±9.5 vs. 51.4±3.7 | PDT | MD | 12 months | BOP, PD, CBL |
| Al-Askar et al. (2021) | 32 (16/16) | 13/3 vs. 10/6 | 65.2±1.3 vs. 62.8±2.5 | PDT | MD | 3 months | PD, PI, CBL, GI |
| Alqahtani et al. (2019) | 98 (49/49) | 49/0 vs. 49/0 | 52.3±2.2 vs. 54.2±2.2 | PDT | MD | 6 months | BOP, PD, PI, CBL |
| Al Rifaiy et al. (2018) | 38 (20/18) | 20/0 vs. 18/0 | 33.6±2.8 vs. 35.4±2.1 | PDT | CHX | 12 weeks | BOP, PD, PI |
| Birang et al. (2017) | 40 (20/20) | 10/10 vs. 10/10 | 36.6±9.7 vs. 36.6±9.7 | PDT | CHX | 3 months | BOP, PD, PI |
| Deeb et al. (2019) | 30 (15/15) | 15/0 vs. 15/0 | 52.6±0.9 vs. 49.2±0.13 | PDT | MD | 12 weeks | BOP, PD, PI |
| Javed et al. (2017) | 54 (28/26) | 28/0 vs. 26/0 | 50.6±0.8 vs. 52.2±0.5 | PDT | MD | 12 weeks | BOP, PD, PI |
| Sánchez-Martos et al. (2017) | 68 (34/34) | 26/8 vs. 14/20 | 59.1±12.1 vs. 54.7±10.5 | DL | MD+CHX | 3 months | BOP, PD, PI |
| Karimi et al. (2016) | 20 (10/10) | 2/8 vs. 2/8 | 52.8±7.33 vs. 52.8±7.33 | PDT | MD | 3 months | BOP, PD, CAL |
| Labban et al. (2021) | 48 (24/24) | 13/11 vs. 11/13 | 47.8±7.2 vs. 50.4±9.3 | PDT | MD | 6 months | BOP, PD, PI, CBL |
| Lerario et al. (2015) | 27 (21/6) | 9/12 vs. 3/3 | 36-67 | DL | MD | 12 months | BOP, PD |
| Persson et al. (2011) | 42 (21/21) | NA | 68.5±6.4 vs. 68.9±12.5 | Er:YAG | MD | 6 months | PD |
| Wang et al. (2017a) | 20 (11/9) | NA | NA | DL | MD | 3 months | PD, PI, BI |
| Wu et al. (2018) | 25 (19/17) | NA | 44 | DL | MD | 24 months | PD, PI, BI |
| He et al. (2018) | 24 (12/12) | NA | 23-60 | DL | MD | 3 months | BOP, PD, PI,GI |
| Wang et al. (2016) | 50 (25/25) | NA | NA | DL | MD | 12 months | PD, PI, BI |
| Bassetti et al. (2014) | 40 (20/20) | 10/10 vs. 10/10 | 59±12.8 vs. 57±11.5 | PDT | MD | 12 months | BOP, PD, CAL |
| Abduljabbar et al. (2016) | 60 (30/30) | 30/0 vs. 30/0 | 50.6±1.4 vs. 51.4±0.6 | PDT | CHX | 6 months | BOP, PD |
| Li et al. (2013) | 30 (15/15) | NA | 47 vs. 47 | PDT | MD | 12 weeks | PD, PI, BI |
| Wang et al. (2017b) | 38 (20/18) | 9/11 vs. 10/8 | 43.1±12.2 vs. 43.0±11.7 | PDT | CHX | 3 months | BOP, PD, PI, BI |
| Wang et al. (2020) | 24 (12/12) | 7/5 vs. 7/5 | 61.5±7.6 vs. 61.3±8.1 | Er:YAG | MD | 6 months | BOP, PD, PI, CAL, GI |
| Altindal et al. (2023) | 40 (20/20) | 12/8 vs. 12/8 | 51.4±10.2 vs. 51.4±10.1 | DL | MD+CHX | 3 months | BOP, PD, PI, GI |
| Aimetti et al. (2019) | 220 (110/110) | 32/78 vs. 39/71 | 58.1±10.1 vs. 56.8±10.2 | DL | MD | 3 months | BOP, PD, PI |
| Li et al. (2023) | 82 (41/41) | 23/18 vs. 25/16 | 46.2±6.3 vs. 45.8±5.5 | DL | CHX | 3 months | PD, PI, BI |
| Li et al. (2022) | 244 (122/122) | 62/60 vs. 58/64 | 68.6±4.2 vs. 69.0±3.4 | Er:YAG | MD+CHX | 6 months | PD, PI, CAL, BI |

Note: DL, Diode Laser; PDT, Photodynamic Therapy; MD, Mechanical Debridement; CHX, Chlorhexidine; BOP, Bleeding on Probing; PD, Probing Depth; PI, Plaque Index; CAL, Clinical Attachment Level; CBL, Crestal Bone Loss; GI, Gingival Index; BI, Bleeding Index, NA, Not Applicable.

**List of included studies:**

1. Schwarz F, Sculean A, Rothamel D, Schwenzer K, Georg T, Becker J. Clinical evaluation of an Er:YAG laser for nonsurgical treatment of peri-implantitis: a pilot study. Clin Oral Implants Res. 2005 Feb;16(1):44-52. doi: 10.1111/j.1600-0501.2004.01051.x. PMID: 15642030.
2. Schwarz F, Bieling K, Bonsmann M, Latz T, Becker J. Nonsurgical treatment of moderate and advanced periimplantitis lesions: a controlled clinical study. Clin Oral Investig. 2006 Dec;10(4):279-88. doi: 10.1007/s00784-006-0070-3. Epub 2006 Sep 13. PMID: 16969659.
3. Schwarz F, Sahm N, Iglhaut G, Becker J. Impact of the method of surface debridement and decontamination on the clinical outcome following combined surgical therapy of peri-implantitis: a randomized controlled clinical study. J Clin Periodontol. 2011 Mar;38(3):276-84. doi: 10.1111/j.1600-051X.2010.01690.x. Epub 2011 Jan 11. PMID: 21219392.
4. Schwarz F, John G, Mainusch S, Sahm N, Becker J. Combined surgical therapy of peri-implantitis evaluating two methods of surface debridement and decontamination. A two-year clinical follow up report. J Clin Periodontol. 2012 Aug;39(8):789-97. doi: 10.1111/j.1600-051X.2012.01867.x. Epub 2012 May 28. PMID: 22639800.
5. Tenore G, Montori A, Mohsen A, Mattarelli G, Palaia G, Romeo U. Evaluation of adjunctive efficacy of diode laser in the treatment of peri-implant mucositis: a randomized clinical trial. Lasers Med Sci. 2020 Aug;35(6):1411-1417. doi: 10.1007/s10103-020-03009-y. Epub 2020 Apr 18. PMID: 32304001.
6. Ahmed P, Bukhari IA, Albaijan R, Sheikh SA, Vohra F. The effectiveness of photodynamic and antibiotic gel therapy as an adjunct to mechanical debridement in the treatment of peri-implantitis among diabetic patients. Photodiagnosis Photodyn Ther. 2020 Dec;32:102077. doi: 10.1016/j.pdpdt.2020.102077. Epub 2020 Nov 4. PMID: 33157330.
7. Arısan V, Karabuda ZC, Arıcı SV, Topçuoğlu N, Külekçi G. A randomized clinical trial of an adjunct diode laser application for the nonsurgical treatment of peri-implantitis. Photomed Laser Surg. 2015 Nov;33(11):547-54. doi: 10.1089/pho.2015.3956. Epub 2015 Sep 18. PMID: 26382562; PMCID: PMC4649765.
8. Wang H, Li W, Zhang D, Li W, Wang Z. Adjunctive photodynamic therapy improves the outcomes of peri-implantitis: a randomized controlled trial. Aust Dent J. 2019 Sep;64(3):256-262. doi: 10.1111/adj.12705. Epub 2019 Jun 21. PMID: 31152567.
9. Al Amri MD, Kellesarian SV, Ahmed A, Al-Kheraif AA, Romanos GE, Javed F. Efficacy of periimplant mechanical debridement with and without adjunct antimicrobial photodynamic therapy in patients with type 2 diabetes mellitus. Photodiagnosis Photodyn Ther. 2016 Jun;14:166-9. doi: 10.1016/j.pdpdt.2016.04.015. Epub 2016 Apr 26. PMID: 27129871.
10. Al-Askar MH, Abdullatif FA, Alshihri AA, Ahmed A, Divakar DD, Almoharib H, Alzoman H. Comparison of photobiomodulation and photodynamic therapy as adjuncts to mechanical debridement for the treatment of peri-implantitis. Technol Health Care. 2022;30(2):389-398. doi: 10.3233/THC-213062. PMID: 34250918.
11. Alqahtani F, Alqhtani N, Alkhtani F, Divakar DD, Al-Kheraif AA, Javed F. Efficacy of mechanical debridement with and without adjunct antimicrobial photodynamic therapy in the treatment of peri-implantitis among moderate cigarette-smokers and waterpipe-users. Photodiagnosis Photodyn Ther. 2019 Dec;28:153-158. doi: 10.1016/j.pdpdt.2019.09.003. Epub 2019 Sep 13. PMID: 31525451.
12. Al Rifaiy MQ, Qutub OA, Alasqah MN, Al-Sowygh ZH, Mokeem SA, Alrahlah A. Effectiveness of adjunctive antimicrobial photodynamic therapy in reducing peri-implant inflammatory response in individuals vaping electronic cigarettes: A randomized controlled clinical trial. Photodiagnosis Photodyn Ther. 2018 Jun;22:132-136. doi: 10.1016/j.pdpdt.2018.03.002. Epub 2018 Mar 15. PMID: 29550362.
13. Birang E, Talebi Ardekani MR, Rajabzadeh M, Sarmadi G, Birang R, Gutknecht N. Evaluation of Effectiveness of Photodynamic Therapy With Low-level Diode Laser in Nonsurgical Treatment of Peri-implantitis. J Lasers Med Sci. 2017 Summer;8(3):136-142. doi: 10.15171/jlms.2017.25. Epub 2017 Jun 27. PMID: 29123634; PMCID: PMC5662503.
14. Deeb MA, Alsahhaf A, Mubaraki SA, Alhamoudi N, Al-Aali KA, Abduljabbar T. Clinical and microbiological outcomes of photodynamic and systemic antimicrobial therapy in smokers with peri-implant inflammation. Photodiagnosis Photodyn Ther. 2020 Mar;29:101587. doi: 10.1016/j.pdpdt.2019.101587. Epub 2019 Nov 2. PMID: 31689510.
15. Javed F, BinShabaib MS, Alharthi SS, Qadri T. Role of mechanical curettage with and without adjunct antimicrobial photodynamic therapy in the treatment of peri-implant mucositis in cigarette smokers: A randomized controlled clinical trial. Photodiagnosis Photodyn Ther. 2017 Jun;18:331-334. doi: 10.1016/j.pdpdt.2017.04.015. Epub 2017 Apr 27. PMID: 28457847.
16. Sánchez-Martos R, Samman A, Bouazza-Juanes K, Díaz-Fernández JM, Arias-Herrera S. Clinical effect of diode laser on peri-implant tissues during non-surgical peri-implant mucositis therapy: Randomized controlled clinical study. J Clin Exp Dent. 2020 Jan 1;12(1):e13-e21. doi: 10.4317/medoral.56424. PMID: 31976039; PMCID: PMC6969958.
17. Karimi MR, Hasani A, Khosroshahian S. Efficacy of Antimicrobial Photodynamic Therapy as an Adjunctive to Mechanical Debridement in the Treatment of Peri-implant Diseases: A Randomized Controlled Clinical Trial. J Lasers Med Sci. 2016 Summer;7(3):139-145. doi: 10.15171/jlms.2016.24. Epub 2016 Jul 18. PMID: 28144432; PMCID: PMC5262478.
18. Labban N, Shibani NA, Al-Kattan R, Alfouzan AF, Binrayes A, Assery MK. Clinical, bacterial, and inflammatory outcomes of indocyanine green-mediated photodynamic therapy for treating periimplantitis among diabetic patients: A randomized controlled clinical trial. Photodiagnosis Photodyn Ther. 2021 Sep;35:102350. doi: 10.1016/j.pdpdt.2021.102350. Epub 2021 May 24. PMID: 34033934.
19. Lerario F, Roncati M, Gariffo A, Attorresi E, Lucchese A, Galanakis A, Palaia G, Romeo U. Non-surgical periodontal treatment of peri-implant diseases with the adjunctive use of diode laser: preliminary clinical study. Lasers Med Sci. 2016 Jan;31(1):1-6. doi: 10.1007/s10103-015-1785-7. Epub 2015 Jul 19. Erratum in: Lasers Med Sci. 2016 Jan;31(1):7. doi: 10.1007/s10103-015-1817-3. PMID: 26188854.
20. Persson GR, Roos-Jansåker AM, Lindahl C, Renvert S. Microbiologic results after non-surgical erbium-doped:yttrium, aluminum, and garnet laser or air-abrasive treatment of peri-implantitis: a randomized clinical trial. J Periodontol. 2011 Sep;82(9):1267-78. doi: 10.1902/jop.2011.100660. Epub 2011 Mar 21. PMID: 21417591.
21. Wang Z, Zou T. Evaluation of the Effect of Semiconductor Laser (GaAlAs) on Peri-Implant Mucositis. J Clin Stomatol (China), 2017, 33(11): 685-687. doi: 10.3969/j.issn.1003-1634.2017.11.015.
22. Wu Y, Jing Z, Zhou Y, Yu J, Zhang Y, Li L. Evaluation of the Efficacy of Diode Laser in Non-Surgical Treatment of Peri-Implantitis. J North Sichuan Med Coll, 2018, 33(1): 44-46. doi: 10.3969/j.issn.1005-3697.2018.01.013.
23. He W. Clinical Observation of Assisted Application of Semiconductor Laser in Patients with Peri-Implant Mucositis. J Clin Stomatol (China), 2018, 34(11): 685-687. doi: 10.3969/j.issn.1003-1634.2018.11.013.
24. Wang Y, Nie Y, Zhang M. Influence of Diode Laser on the Treatment Effect of Peri-Implantitis. Gen J Stomatol, 2016, 3(3): 123-126. doi: 10.16269/j.cnki.cn11-9337/r.2016.03.081.
25. Bassetti M, Schär D, Wicki B, Eick S, Ramseier CA, Arweiler NB, Sculean A, Salvi GE. Anti-infective therapy of peri-implantitis with adjunctive local drug delivery or photodynamic therapy: 12-month outcomes of a randomized controlled clinical trial. Clin Oral Implants Res. 2014 Mar;25(3):279-287. doi: 10.1111/clr.12155. Epub 2013 Apr 8. PMID: 23560645.
26. Abduljabbar T. Effect of mechanical debridement with and without adjunct antimicrobial photodynamic therapy in the treatment of peri-implant diseases in prediabetic patients. Photodiagnosis Photodyn Ther. 2017 Mar;17:9-12. doi: 10.1016/j.pdpdt.2016.10.011. Epub 2016 Oct 29. PMID: 27989865.
27. Li Y, Fa Y, Yang Y, Cai X, Li G. Treatment of Peri-implantitis with Photodynamic Therapy. J Pract Stomatol. 2013 Nov;29(6):848-850. doi: 10.3969/j.issn.1001-3733.2013.06.022.
28. Wang Y, Ma Y, Liu X, Guo L. Short-term Clinical Effects on the Treatment of Peri-implantitis with Photodynamic Therapy. Henan Medical Research. 2017 Aug;26(16):2890-2892. doi: 10.3969/j.issn.1004-437X.2017.16.003.
29. Wang CW, Ashnagar S, Gianfilippo RD, Arnett M, Kinney J, Wang HL. Laser-assisted regenerative surgical therapy for peri-implantitis: A randomized controlled clinical trial. J Periodontol. 2021 Mar;92(3):378-388. doi: 10.1002/JPER.20-0040. Epub 2020 Aug 25. PMID: 32761810.
30. Altindal D, Alkan EA, Calisir M. Evaluation of the effectiveness of diode laser therapy in conjunction with nonsurgical treatment of peri-implantitis. J Periodontal Implant Sci. 2023 Oct;53(5):376-387. doi: 10.5051/jpis.2203140157. Epub 2023 Feb 3. PMID: 37038829; PMCID: PMC10627738.
31. Aimetti M, Mariani GM, Ferrarotti F, Ercoli E, Liu CC, Romano F. Adjunctive efficacy of diode laser in the treatment of peri-implant mucositis with mechanical therapy: A randomized clinical trial. Clin Oral Implants Res. 2019 May;30(5):429-438. doi: 10.1111/clr.13428. Epub 2019 Apr 16. PMID: 30933384.
32. Li Suna, Song Wenshang, Liu Zilue, Cai Shixin, Tang Xiaoli. Effect of Semiconductor Laser on the Treatment of Mild to Moderate Peri-Implantitis. Hengshui People's Hospital, Hebei, China. doi: 10.11816/cn.ni.2023-222229.
33. Li Shihui, He Zhiliang, Ma Huiying, Huang Xianbo, Li Chunjia, Cui Yanyan. Clinical Efficacy of Erbium Laser Therapy for the Treatment of Peri-Implantitis in the Elderly.Chengde Central Hospital, Hebei, China. doi: 10.3969/j.issn.1005-9202.2022.07.042.
