# Supplementary material for: Comparative effectiveness of novel laser-assisted technologies for treating peri-implantitis: A network meta-analysis of randomized controlled trials
Source: Medicine (Baltimore). 2026 Oct 30;105(44):e51256. doi: 10.1097/MD.0000000000051256 (PMC13727279; doi:10.1097/MD.0000000000051256)
Supplement: Supplementary file 6 [file medi-105-e51256-s013.docx]

# Supplementary 7: The risk of bias assessment for the individual included studies

| **Study** | Randomization process | Deviations from intended interventions | Missing outcome data | Measurement of the outcome | Selection of the reported result | Overall Bias |
| --- | --- | --- | --- | --- | --- | --- |
| Schwarz et al. (2005) | Low | Some concerns | Low | Low | Low | Some concerns |
| Schwarz et al. (2006) | Some concerns | Low | Low | Low | Low | Some concerns |
| Schwarz et al. (2011) | Some concerns | Low | Low | Low | Low | Some concerns |
| Schwarz et al. (2012) | Low | Low | High | Low | Low | High |
| Tenore et al. (2020) | Low | Low | Low | Low | Some concerns | Some concerns |
| Ahmed et al. (2020) | Low | Low | Low | Low | Low | Low |
| Arısan et al. (2015) | Low | Low | Low | Low | Low | Low |
| Wang et al. (2019) | Some concerns | Low | Low | Low | Low | Some concerns |
| Amri et al. (2016) | Low | Low | Some concerns | Low | Low | Some concerns |
| Al-Askar et al. (2021) | Low | Low | Low | Low | Low | Low |
| Alqahtani et al. (2019) | Low | Low | Low | Low | Low | Low |
| Al Rifaiy et al. (2018) | Some concerns | Some concerns | Some concerns | Low | Low | Some concerns |
| Birang et al. (2017) | Low | Low | Low | Low | Low | Low |
| Deeb et al. (2019) | Low | Low | Low | Low | Low | Low |
| Javed et al. (2017) | Some concerns | Low | Low | Low | Low | Some concerns |
| Sánchez-Martos et al. (2017) | Some concerns | Low | Low | Low | Low | Some concerns |
| Karimi et al. (2016) | Low | Low | Some concerns | Low | Low | Some concerns |
| Labban et al. (2021) | Low | Low | Low | Low | Low | Low |
| Lerario et al. (2015) | Low | Low | Low | Low | Low | Low |
| Persson et al. (2011) | Some concerns | Some concerns | Some concerns | Low | Low | Some concerns |
| Wang et al. (2017a) | Low | Low | Low | Low | Low | Low |
| Wu et al. (2018) | Low | Low | Low | Low | Low | Low |
| He et al. (2018) | Some concerns | Low | Low | Low | Low | Some concerns |
| Wang et al. (2016) | Low | Low | High | Low | Low | High |
| Bassetti et al. (2014) | Low | Low | Low | Low | Some concerns | Some concerns |
| Abduljabbar et al. (2016) | Low | Low | Low | Low | Low | Low |
| Li et al. (2013) | Low | Low | Low | Low | Low | Low |
| Wang et al. (2017b) | Low | Low | Low | Low | Low | Low |
| Wang et al. (2020) | Low | Low | Low | Low | Low | Low |
| Altindal et al. (2023) | Some concerns | Low | Low | Low | Low | Some concerns |
| Aimetti et al. (2019) | Some concerns | Low | Low | Low | Low | Some concerns |
| Li et al. (2023) | Low | Low | Low | Low | Some concerns | Some concerns |
| Li et al. (2022) | Some concerns | Low | Low | Low | Low | Some concerns |
